# Transcriptional and Epigenetic Response to Sedentary Behavior and Physical Activity in Children and Adolescents: A Systematic Review

**DOI:** 10.3389/fped.2022.917152

**Published:** 2022-06-24

**Authors:** Abel Plaza-Florido, Inmaculada Pérez-Prieto, Pablo Molina-Garcia, Shlomit Radom-Aizik, Francisco B. Ortega, Signe Altmäe

**Affiliations:** ^1^Department of Physical and Sports Education, Faculty of Sport Sciences, PROFITH “PROmoting FITness and Health Through Physical Activity” Research Group, Sport and Health University Research Institute (iMUDS), University of Granada, Granada, Spain; ^2^Department of Biochemistry and Molecular Biology, Faculty of Sciences, University of Granada, Granada, Spain; ^3^Instituto de Investigación Biosanitaria (ibs.GRANADA), Granada, Spain; ^4^Physical Medicine and Rehabilitation Service, Virgen de las Nieves University Hospital, Granada, Spain; ^5^Pediatric Exercise and Genomics Research Center, UC Irvine School of Medicine, Irvine, CA, United States; ^6^Faculty of Sport and Health Sciences, University of Jyväskylä, Jyväskylä, Finland; ^7^Department of Biosciences and Nutrition, Karolinska Institutet, Huddinge, Sweden; ^8^Division of Obstetrics and Gynecology, CLINTEC, Karolinska Institutet, Stockholm, Sweden; ^9^Competence Centre on Health Technologies, Tartu, Estonia

**Keywords:** exercise, methylation, omics, physical fitness, RNA-seq, epigenomics

## Abstract

**Background:**

The links of sedentary behavior and physical activity with health outcomes in children and adolescents is well known. However, the molecular mechanisms involved are poorly understood. We aimed to synthesize the current knowledge of the association of sedentary behavior and physical activity (acute and chronic effects) with gene expression and epigenetic modifications in children and adolescents.

**Methods:**

PubMed, Web of Science, and Scopus databases were systematically searched until April 2022. A total of 15 articles were eligible for this review. The risk of bias assessment was performed using the Joanna Briggs Institute Critical Appraisal Tool for Systematic Reviews and/or a modified version of the Downs and Black checklist.

**Results:**

Thirteen studies used candidate gene approach, while only 2 studies performed high-throughput analyses. The candidate genes significantly linked to sedentary behavior or physical activity were: *FOXP3*, *HSD11B2*, *IL-10, TNF-*α, *ADRB2, VEGF*, *HSP70*, *SOX*, and *GPX*. Non-coding Ribonucleic acids (RNAs) regulated by sedentary behavior or physical activity were: miRNA-222, miRNA-146^a^, miRNA-16, miRNA-126, miR-320^a^, and long non-coding RNA MALAT1. These molecules are involved in inflammation, immune function, angiogenic process, and cardiovascular disease. Transcriptomics analyses detected thousands of genes that were altered following an acute bout of physical activity and are linked to gene pathways related to immune function, apoptosis, and metabolic diseases.

**Conclusion:**

The evidence found to date is rather limited. Multidisciplinary studies are essential to characterize the molecular mechanisms in response to sedentary behavior and physical activity in the pediatric population. Larger cohorts and randomized controlled trials, in combination with multi-omics analyses, may provide the necessary data to bring the field forward.

**Systematic Review Registration:**

[www.ClinicalTrials.gov], identifier [CRD42021235431].

## Introduction

Some global estimations point out that 81% of children and adolescents do not meet current physical activity guidelines (≥ 60 min of daily moderate to vigorous intensity physical activity) (1, 2). One study reported how physical activity levels decreased between 1995 and 2017, especially in adolescents (3). Importantly, the negative impact of sedentary behavior and lack of physical activity on different health-related outcomes (cardiometabolic risk factors, brain health, among others) in children and adolescents is well known (2, 4–7). However, little is known about the molecular mechanisms underlying the effects of sedentary behavior and physical activity (acute or chronic effects) on health in children and adolescents. Sedentary behavior is considered any behavior that implies energy expenditure ≤ 1.5 metabolic equivalents (METs) while sitting, reclining or laying (8–10). Physical activity is defined as any body movement that requires energy expenditure higher than in resting conditions (8–10).

At the single-gene approach, many of the studies have reported how physical activity modulates the association between candidate gene sequence variants (e.g., single nucleotide polymorphisms) and cardiometabolic risk factors (e.g., blood pressure, body fat, among others) in pediatric population (11–14). Epigenetic modifications such as DNA methylation, histone acetylation and microRNAs (miRNAs) are known to be modulated by lifestyle factors such as sedentary behavior and/or physical activity (15, 16), should be further studied to understand their effect on gene expression. Interestingly, non-coding RNAs such as microRNAs (miRNAs) are considered one of the novel molecular biomarkers in the physical activity-mediated interactions, which might modulate protein and metabolite expression at the post-transcriptional level by binding to coding messenger RNAs (mRNAs) (17, 18). Therefore, transcription (i.e., gene expression) and translation (i.e., protein expression) are influenced by epigenetic modifications, which might have an impact on phenotype and physiological functions (15).

Technological advances in molecular biology, such as high through-put omics platforms allow exact and simultaneous examinations of thousands of genes, proteins and metabolites at the genome-wide level (19). Physical activity can alter gene pathways involved in immune response, inflammation, and cardiovascular signaling (STAT3 pathway, VEGF signaling, Chemokine signaling, NF-κB, and MAPK-pathways, among others) in blood cells of adults (20–22). However, these molecular mechanisms are still poorly understood in the pediatric population, and comprehensive overview on the topic is lacking. This systematic review aimed to provide a summary of the current literature on the effects of sedentary behavior and physical activity (acute and chronic effects) on gene expression and epigenetic mechanisms in children and adolescents.

## Methods

For this systematic review, we used the Preferred Reporting Items for Systematic Review and Meta-Analysis (PRISMA) 2020 guidelines (23). The review protocol was registered in the International Prospective Register of Systematic Reviews (PROSPERO) with the reference number: CRD42021235431.

### Search Strategy and Eligibility Criteria

A systematic search was conducted in PubMed, Web of Science, and Scopus databases up to 05 April 2022. Detailed search strategy is available in Supplementary Table 1. Search terms were selected based on the exercise and molecular biology concepts of interest. Table 1 lists the definitions of the main molecular biology-related terms used in this systematic review, for those researchers, exercise physiologists, or clinicians less familiarized with molecular biology-related terminology. The inclusion criteria were: (1) children and/or adolescents aged ≤ 18years; (2) observational articles (cross-sectional or longitudinal) that study the relationship of sedentary behavior and/or physical activity with gene expression and epigenetics modifications (both candidate gene and high-throughput approaches); (3) articles that report the acute and/or chronic effects of physical activity (e.g., intervention studies/trials and cross-over study designs) on gene expression and/or epigenetics modifications (both candidate gene and high-throughput approaches). The exclusion criteria were defined as follows: (1) studies that reported the acute and/or chronic effects of physical activity combined with other lifestyle interventions such as nutrition interventions, probiotic or prebiotic supplementation or caloric restriction, so that the independent effect of sedentary behavior or physical activity could not be extracted; (2) articles written in any language other than English or Spanish; (3) letters to the editor, editorials, meeting abstracts, study protocols, or reviews.

**TABLE 1:** Definition of the main molecular biology-related terms used in this systematic review.

Term	Definition
mRNA	Messenger RNA (mRNA) carries the genetic information from nucleus to ribosomes necessary to synthesize proteins. Gene expression analysis is based on analysing mRNA molecules.
Epigenetics	Epigenetic modifications (i.e., DNA methylation, histone acetylation) that act on DNA structure. These mechanisms can activate or repress transcription (i.e., gene expression). miRNA is also considered a form of epigenetic regulation, see description below.
CpG site	DNA region prone to methylation where a cytosine nucleotide is followed by a guanine nucleotide linked by a phosphate group.
DNA methylation	One of the most studied epigenetic modifications that consists in adding a methyl group to C nucleotide in DNA.
Histone acetylation Microarray	Epigenetic modification that involves the addition of an acetyl group to the histone proteins. Microarray is a technology that detects the expression levels of thousands of genes at the same time. Briefly, thousands of genetic sequences are located on a chip, and based on the complementary sequences of the transcripts in a biological sample the hybridization takes place, allowing the detection of gene expression levels.
miRNA	Non-coding micro RNA (miRNA) molecule that is small in length, 18–24 pair of bases. These small RNA molecules are able to regulate gene expression by influencing the half-life of the mRNA or it’s availability for translation.
omics	Refers to analyses of entire set of molecules such as proteins (i.e., proteomics), metabolites (i.e., metabolomics), DNA sequence variants (i.e., genomics), mRNA expression (i.e., transcriptomics), or DNA methylation profile (i.e., epigenomics) within the sample.
RNA-seq	RNA sequencing technique to quantity the gene expression profile (i.e., transcriptome) in a biological sample.
qPCR Transcriptome	Laboratory technique based on polymerase chain reaction (PCR), which is widely used in molecular biology to amplify a specific nucleic acid sequence and obtain millions to billions of copies. This technique is able to quantify gene expression levels. Analysis of transcripts (typically mRNA molecules) in order to assess the gene expression levels. Both microarray and RNA-seq approaches are used. The difference between these methods is that in the array a set of possible genes is defined by the set of probes that are present, while RNA-seq allows detection of known and unknown genes.

*RNA, Ribonucleic acid; mRNAs, messenger ribonucleic acids; miRNA, micro-RNA DNA, Deoxyribonucleic acid; CpG, Cytosine-phosphate-Guanine; qPCR, quantitative polymerase chain reaction; RNA-seq, RNA sequencing.*

### Study Selection and Data Extraction

The relevant articles were identified by two researchers (AP-F and IP-P) screening by the title and abstract using the Covidence tool.^1^ Then, full-text articles were reviewed by the same researchers to determine final eligibility. Two researchers (AP-F and IP-P) discussed conflictive articles until a common consensus was reached. The data extraction, performed by one researcher (IP-P) and double checked by one independent researcher (AP-F), included the following information: (1) study design; (2) sample characteristics (i.e., size, gender, age, and ethnicity/race); (3) characteristics of the exposure (i.e., sedentary behavior or physical activity); (4) tissue; (5) dependent outcome (i.e., gene expression or epigenetics); (6) main findings.

### Risk of Bias Assessment

Risk of bias for each eligible article was performed by two researchers (AP-F and IP-P) using the Joanna Briggs Institute Critical Appraisal Tool for Systematic Reviews (24). The different checklists included in this tool are specific for each study design (e.g., cross-sectional studies, non-randomized controlled trials). The items in each checklist had 4 possible answers: “yes” (criterion met), “no” (criterion not met), “unclear” or “not applicable.” Particularly, the checklists used were those for cross-sectional studies and non-randomized controlled trials, which include eight and nine items, respectively. For acute physical activity studies we used a modified version of the Downs and Black checklist (25). This checklist contains 17 questions and was previously adapted for the risk of bias assessment of articles that reported the acute effects of physical activity on bone biomarkers (26). The quality score per item (%) was calculated by dividing the number of studies that met the quality criteria in one specific item (e.g., answer as yes in item number 1) by the total number of studies (e.g., 5 cross-sectional studies). The lower is the score in each item (expressed in %) the lower is the quality of that item and therefore the higher is the bias in that item. As an example, a 40% score in the item number 1 and a 100% in the number 2 is indicating a lower quality and higher bias in item 1 compared to item number 2.

## Results

**FIGURE 1:**
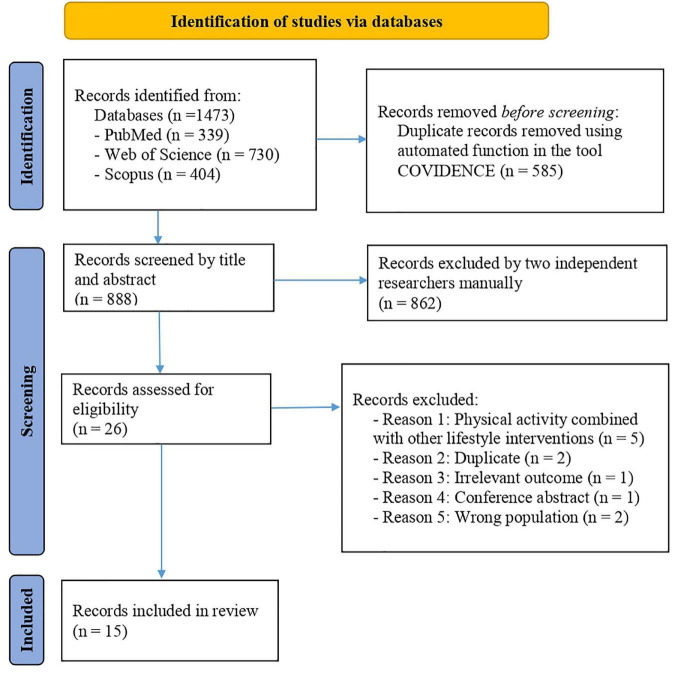
Study selection process based on the Preferred Reporting Items for Systematic Reviews and Meta-Analyses (PRISMA) 2020 flow diagram.

**FIGURE 2:**
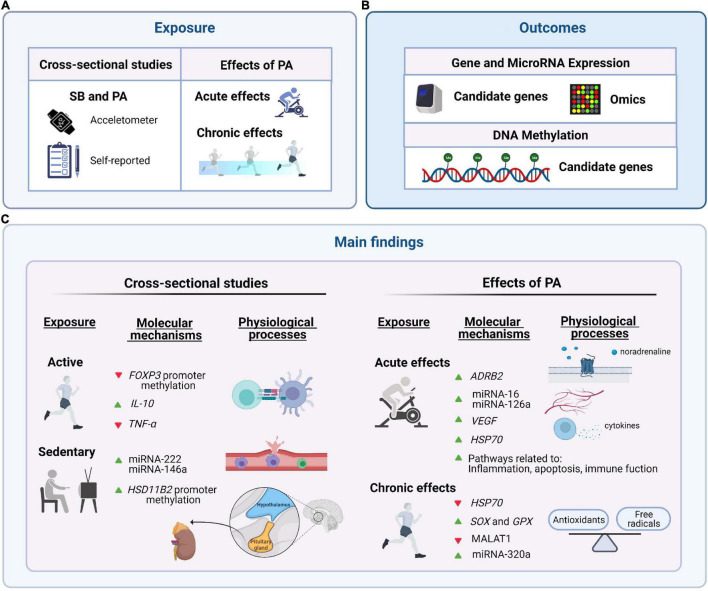
Summary of the main candidate genes and gene pathways related to sedentary behavior (SB) and physical activity (PA) (i.e., acute and chronic effects) in the pediatric population. **(A)** Exposure: SB and PA (acute and chronic effects). **(B)** Outcomes: gene expression and epigenetics (candidate genes and high-throughput transcriptomics analyses). **(C)** Main findings: relevant genes identified in our systematic review. Green arrows reflect up-regulation and red arrows down-regulation. This figure was created with BioRender.com.

PRISMA checklist 2020 shows the appropriateness of the methods performed in our systematic review (Supplementary Tables 2, 3). Figure 1 illustrates the PRISMA 2020 flow diagram for the selection process of the studies: a total of 1,473 articles were included from the three databases, and after removing the duplicates and non-eligible studies, 15 articles remained eligible for this review (6 cross-sectional articles, 5 studies reported the acute effects of physical activity, and 5 articles showed the chronic effects of physical activity). The sample size ranged from 12 to 369 participants (27–41). The age of participants ranged from 9 to 18 years old (27–41). Thirteen studies used blood samples (27, 29–32, 34–41) while 2 saliva (33) and buccal swabs (28) respectively. Regarding disease, four studies included children with obesity (27, 34, 38, 41) and 1 study children with HIV infection (29). Concerning countries/regions, 4 studies were performed in the United States of America (28, 31, 36, 37), 2 in Brazil (30, 38), 4 in Europe (27, 33, 35, 39), 3 in Asia (32, 34, 41), 1 in Mexico (40), and 1 in India (29). All the relevant information extracted from each article is presented in Table 2. In addition, a graphical summary of the mains results is presented in Figure 2. Specific genes and related pathways found in the studies are interpreted and discussed in the context of existing knowledge in the Discussion section.

**TABLE 2:** Summary of study characteristics of articles included in this review.

Sedentary behavior and physical activity: cross-sectional evidence						
**References**	**Study design**	**Target population [Sample size (N)]; Sex (boys %); Age (SD or range in years); Ethnicity/Race (%)**	**Characteristics of the exposure (SB, PA) or PA intervention**	**Tissue**	**Dependent outcome and analytical method**	**Main findings**
Wu et al. (34)	Cross-sectional	Group 1: Children with obesity (*N* = 59); Boys + Girls (45.8%); 13.8 ± 3.0 y; Chinese (100%) Group 2: Normal-weight children (*N* = 39); Boys + Girls (61.5%); 10.3 ± 1.1 y; Chinese (100%)	SB and PA across 6 months (questionnaire completed by parents or guardians)	Leukocytes	DNA methylation at *FAIM2* promoter (Sequenom MassARRAY platform)	Differentially methylation levels at *FAIM2* promoter between obese and normal-weight children according to SB and PA levels. Results were not significant after multiple hypothesis testing corrections
Lovinsky-Desir et al. (28)	Cross-sectional	Group 1: Active children (*N* = 77); Boys + Girls (45%); 12.2 y (9.2–14.0 y); Hispanic (60%), African American (40%) Group 2: Non-active children (*N* = 58); Boys + Girls (55%); 12.7 y (10.5–14.0 y); Hispanic (72%), African American (28%)	PA across 6 days (accelerometer on the non-dominant wrist)	Buccal swabs (squamous epithelial cells)	DNA methylation at *FOXP3* promoter (pyrosequencing) and gene expression	Active children had lower *FOXP3* promoter methylation compared to Non-active children exposed to high air pollutant black carbon concentrations. No significant association was reported between *FOXP3* promoter methylation and gene expression
Vriens et al. (33)	Cross-sectional	Children with normal-weight 70%, overweight 12.5%, and underweight 17.5% (*N* = 80); Boys + Girls (46.3%); 10.44 ± 0.97 y; Caucasian (91.3%)	SB and PA across ∼2 years (out-of-school sport activities and screen time use questionnaires filled out by the parents)	Extracellular fraction of saliva	Expression levels of miRNA-222 and miRNA-146a (qPCR)	SB, represented by screen time use, was positively associated with miRNA-222 and miRNA-146a levels. PA was not significantly associated with either miRNA-222 or miRNA-146a
Wu et al. (40)	Cross-sectional	Adolescents (*N* = 369); Boys + Girls (47.2%); 14.22 ± 1.99 y for boys/13.95 ± 2.04 y for girls; Mexican (100%)	SB and PA across 7 days (accelerometer on the non-dominant wrist)	Leukocytes	DNA methylation at *PPARA*, *H19*, *LINE-1*, and *HSD11B2* (pyrosequencing)	Substituting 30-min of vigorous PA for 30-min of SB daily was associated with higher methylation at *HSD11B2* promoter in boys
Gopalan et al. (29)^a^	Cross-sectional	Group 1: Exercisers (*N* = 20); Boys + Girls with HIV infection (75%); 10.5 y; Indian (100%) Group 2: Non-exercisers (*N* = 20); Boys + Girls with HIV infection (44.4%); 12.5 y; Indian (100%)	Children who practiced 20–45 min/day, 4 times per week from year 0 to year 2 were categorized as “exercisers” (physical activity questionnaire suited for Indian children)	PBMC	*IL-2* and *BDNF* gene expression (qPCR)	The gene expression of *IL-2* and *BDNF* was not significantly different between exercisers and non-exercisers groups
Dos Santos Haber et al. (30)	Cross-sectional	Children and adolescents (*N* = 108) divided into 4 groups (type I diabetes with ketoacidosis; decompensated type I diabetes; Compensated type I diabetes and healthy control); Boys + girls (NR); 10–18 years old; NR	Frequency and duration of PA activities recorded during the last 3 months by questionnaires. Children were classified as low active (<150 min/week), active (150–250 min/week), and very active (>250 min/week)	Blood samples	*IL-10* and *TNF-*α (qPCR)	A higher PA level (very active compared to active and control groups) was associated with increased *IL-10* and decreased *TNF-*α expression in children with type I diabetes/ketoacidosis and decompensated type I diabetes

**Acute effects of physical activity**						

Radom-Aizik et al. (37)	Within-subjects experiment	Group 1: Early-pubertal boys (*N* = 10); Boys; 10.5 ± 0.4 y; NR Group 2: Late-pubertal boys (*N* = 10); Boys; 17.4 ± 0.4 y; NR	Cycle ergometer test, 10 × 2 min bouts, the work rate was individualized for each boy (∼90% of HR_*peak*_) with 1-min rest intervals	PBMC	Microarray gene expression (Affymetrix U133 + 2 arrays)	A single bout of PA induced changes in PBMC gene expression in both groups, particularly 1,246 genes (517 up, 729 down) in late-pubertal boys and 109 (79 up, 30 down) in early pubertal boys. 13 gene pathways involved in immune function and type I diabetes, were altered by acute PA in both early- and late-pubertal boys
Radom-Aizik et al. (36)	Within-subjects experiment	Group 1: Early-pubertal girls (*N* = 10); Girls; 10.0 ± 0.3 y; NR Group 2: Late-pubertal girls (*N* = 10); Girls; 16.1 ± 0.4 y; NR	Cycle ergometer test, 10 × 2 min bouts, the work rate was individualized for each girl (∼90% of HR_*peak*_) with 1-min rest intervals	PBMC	Microarray gene Expression (Affymetrix U133 + 2 arrays)	A single bout of PA induced changes in PBMC gene expression in both groups, particularly, 877 genes (611 up, 266 down) in late-pubertal girls and 1,320 (829 up, 491 down) in early-pubertal girls. 5 gene pathways related to inflammation, stress, and apoptosis, were altered by acute PA in both early- and late-pubertal girls
Kochanska-Dziurowicz et al. (39)	Within-subjects experiment	Youth ice hockey players (*N* = 19); Boys; 17.1 ± 0.5 y; Polish (100%)	Cycle ergometer test until voluntary exhaustion (starting with 1.0 W⋅kg^–1^ load and increasing the intensity by 0.5 W⋅kg^–1^ each 3 min)	PBMC	*ADRB2* and *ACTB* gene expression (qPCR)	*ADRB2* and *ACTB* (internal control) gene expression increased in 74% of players after the PA test
Kilian et al. (35)	Cross-over experiment	Competitive young cyclists (*N* = 12); Boys; 14.4 ± 0.8 y; NR	Session 1: HIIT, 4 × 4 min at 90–95% PPO with 3-min active recovery intervals at 45% PPO Session 2: HVT, 90 min at 60% PPO	Capillary blood samples	Expression levels of miRNA-16, miRNA-21, miRNA-126, and VEGF mRNA (qPCR)	HVT significantly increased miRNA-16 and miRNA-126 during and after the PA test, whereas HIIT showed no significant influence on the miRNAs. VEGF gene expression significantly increased during and after HIIT and HVT
Lu et al. (31)^b^	Within-subjects experiment	Group 1: Asthmatics adolescents (*N* = 12); Boys + Girls (33.3%); 15.7 y (14.0–17.0 y); White (50%), Asian (42%), more than one ethnicity (1%) Group 2: Healthy adolescents (*N* = 14); Boys + Girls (57.1%); 15.0 y (14.0–17.0 y); White (71%), Asian (21%), more than one ethnicity (7%)	Acute effects of PA: Cycle ergometer test, 10 × 2min at ∼75% of VO_2p*eak*_ with 1-min rest intervals Chronic effects of PA: 8-weeks, 3 days/week (1 h-session)	PBMC	*GR (NR3C1)*, *GR*β, *HSP70*, *TGF*β*1*, and *TGF*β*2* gene expression (qPCR)	No effect on PBMC gene expression of *NR3C1*, *GR*β, *TGF*β*1*, and *TGF*β*2* in both healthy and asthmatic adolescents. In addition, *HSP70* gene expression was increased after acute PA while was decreased after chronic PA intervention

**Chronic effects of physical activity**						

Woo et al. (32)^c^	Non-randomized controlled trial	Group 1: Children with overweight (*N* = 20); Boys; 11.30 ± 1.17 y; Korean (100%) Group 2: Normal-weight children (*N* = 19); Boys; 11.32 ± 1.06 y; Korean (100%)	12-weeks PA intervention. The characteristics of the PA intervention were unclear (i.e., intensity, frequency, among others)	PBMC	*SOD* and *GPX* gene expression (qPCR)	*SOD* and *GPX* gene expression was up-regulated after 12-weeks of PA in both groups. In addition, *SOD* and *GPX* gene expression was up-regulated after 24-weeks of PA in children with overweight
Blüher et al. (27)	Non-randomized controlled trial	Adolescents with overweight/obesity (*N* = 28); Boys + Girls (46.5%); 15.5 ± 1.4 y; NR	HIIT, 6-months, 2 sessions/week, 60 min/session at 80–95% HR_*max*_ with active breaks at 50–60% of HR_*max*_	Blood samples	DNA methylation at *RALBP1* (pyrosequencing)	No significant changes in levels of methylation at *RALBP1* were observed after 6-months of PA intervention in children with overweight/obesity
Zhao et al. (41)	Non-randomized controlled trial	Children and adolescents with obesity (PA intervention group *N* = 40; control group *N* = 20); Boys + Girls (68.3%); 8–16 y; NR	12-weeks PA intervention. Frequency of 5 sessions/week, 50 min each session, intensity 60–70% of HR_*max*_	Blood samples	Long non-coding RNA MALAT1 and miR-320a expression (qPCR)	PA intervention decreased MALAT1 and increased miR-320a expression
De Souza E Silva et al. (38)	Non-randomized controlled trial	Children and adolescents with overweight/obesity (PA intervention group *N* = 17; control group *N* = 18); Boys + Girls (53.0%); 10–16 y; Euro-Brazilian (self-reported)	12-weeks PA intervention (indoor cycling), 3 sessions/week (60 min/session)	Blood samples	*ADRB2* gene expression (qPCR)	No significant changes in levels of *ADRB2* expression were reported after 12-weeks of PA intervention in children with overweight/obesity.

*^a^The study design was retrospective cohort study. However, gene expression analysis was performed only at year 2 (cross-sectional) between “exercisers” and “non-exercisers.”*

*^b^This study reported acute and chronic effects of physical activity in gene expression.*

*^c^To assess the detraining effect in SOD and GPX gene expression, children with overweight were divided (at the end of 12-weeks of physical activity program) into an overweight training group (i.e., in addition, performed 12-weeks of physical activity) and an overweight detraining group (i.e., in addition, performed 12-weeks of detraining). Boys (B); Girls (G); Glucocorticoid receptor (GR); High intensity interval training (HIIT); High volume session (HVT); Kilogram (Kg); Kilometer (Km); Maximal heart rate (HR_max_); Micro-RNA (miRNA); Minutes (min); Not reported (NR); Peak heart rate (HR_peak_); Peak oxygen consumption (VO_2_peak); Peak power output (PPO); Peripheral blood mononuclear cells (PBMC); Physical Activity (PA); quantitative polymerase chain reaction (qPCR); Sedentary behavior (SB); Wattios (W); Years (Y).*

### Sedentary Behavior and Physical Activity: Cross-Sectional Studies

Six cross-sectional studies out of the fifteen articles presented in Table 2 reported the associations of sedentary behavior or physical activity with DNA methylation or gene expression using candidate gene analyses (28–30, 33, 34, 40). Four studies reported significant associations (28, 30, 33, 40), while one study showed significant associations that disappeared after multiple hypothesis testing corrections (34). Also, one study did not report significant associations between physical activity and gene expression of the 2 candidate genes (interleukin-2 and brain-derived neurotrophic factor) that were tested (29). Sedentary behavior and physical activity were reported by questionnaires in four studies, with an assessment time ranging from 6 months to 2 years (29, 30, 33, 34). Two studies measured physical activity and/or sedentary behavior objectively using accelerometers on the non-dominant wrist for 6 days (28, 40). Gene expression and DNA methylation levels were obtained from white blood cells in three studies (29, 34, 40), whole blood in one study (30), and saliva in two studies (28, 33). All observational studies/gene expression analyses were performed at cross-sectional level, while longitudinal studies/analyses were not performed.

### Acute Effects of Physical Activity

Five out of the twelve articles presented in Table 2 reported significant effects of acute bout of physical activity on gene expression (31, 35–37, 39). Among the five studies, three reported the effects of acute bout of physical activity using candidate gene analyses (i.e., mRNA or miRNA expression) (31, 35, 39), while two studies performed high-throughput transcriptomics analyses using microarrays (36, 37). Four studies used circulating peripheral blood mononuclear cells (PBMCs) to quantify gene expression (31, 36, 37, 39), while one study used capillary blood samples from the earlobe (35).

### Chronic Effects of Physical Activity

Three out of the fifteen articles presented in Table 2 reported significant effects of chronic physical activity on gene expression (31, 32, 41), while two studies did not detect any effect of chronic physical activity on DNA methylation (27) and gene expression (38) respectively, using candidate gene analyses (*RALBP1* and *ADRB2* genes). The duration of the physical activity interventions was 12-week (27, 32, 38, 41) and 8-week (31). In two studies, the intensity of physical activity was unclear (32, 38), while in three studies the intensity was well-defined using specific% of maximal heart rate (HRmax) during physical activity and breaks (27, 31, 41). The five studies used blood samples to quantify gene expression or DNA methylation (27, 31, 32, 38, 41).

### Risk of Bias Assessment

Among the 6 cross-sectional studies the items 1 and 3 obtained the lowest bias per item score. These items reflect that the sample inclusion criteria were not clearly defined, and the exposure (i.e., sedentary behavior and physical activity) was not measured in a valid a reliable way (Supplementary Table 4). Concerning the 5 studies that reported the effects of acute physical activity, item 16 obtained the lowest bias per item score. This reflects that most acute physical activity studies did not consider unusual activity or nutritional factors the day before performing the physical activity test (Supplementary Table 5). Regarding the chronic effects of physical activity, item 4 obtained the lowest bias per item score that reflects the absence of a control group not exposed to the interest intervention (i.e., long-term physical activity intervention) (Supplementary Table 6).

## Discussion

This study aimed to provide current knowledge on the effect of sedentary behavior and physical activity on gene expression and epigenetic mechanisms in the pediatric population. The main findings and gaps identified by this systematic review in children and adolescents were: (1) there is very limited information of the molecular mechanisms of sedentary behavior and/or physical activity on gene expression and its regulation in pediatric population; (2) most of the studies showed that sedentary behavior and physical activity (acute and chronic effects) alter gene and MicroRNA expression, and DNA methylation of candidate genes related to obesity, asthma, immune function, and cardiovascular disease; (3) the studies are hardly comparable due to different candidate genes selected, characteristics of the exposure, health and training status of the participants, and study designs; (4) only two studies performed high-throughput transcriptomics analyses and detected thousands of genes differentially altered by acute bout of physical activity in boys and girls at different pubertal stages (36, 37). The relatively small number of studies, the heterogeneity in the methodology, different study designs, and most of the studies were performed in Europe and/or the United States of America (8/15) limit the extrapolation of our findings to the general pediatric population. Studies using high-throughput techniques (i.e., sequencing) and longitudinal study approach and/or randomized controlled trials on bigger cohorts are lacking in children and adolescents.

### Sedentary Behavior and Physical Activity: Cross-Sectional Studies

The genes and/or miRNAs selected by the five cross-sectional studies detected in our review, were related to obesity (*FAIM2*) (42), cardiac hypertrophy, angiogenesis and inflammation (miRNA-222 and miRNA-146a) (33, 43, 44), signaling molecule in the immune system (*IL-2*) (29), brain health (*BDNF*) (29), T regulatory cells differentiation and function (*FOXP3*) (45), and stress/cortisol metabolism (*HSD11B2*) (46). Wu et al. reported differential methylation levels at several CpG sites at the *FAIM2* promoter region between obese and normal-weight children according to sedentary behavior and physical activity levels assessed by questionnaires (physical activity threshold 150 min/week) (34). FAIM2 is involved in apoptosis and neurogenesis and is also influenced by food restriction in rodents (47, 48). Some studies reported that polymorphisms near *FAIM2* as well as promoter methylation levels might be associated with obesity (49–51). Thus, *FAIM2* promoter methylation levels could be influenced by sedentary behavior and physical activity affecting health status in children with obesity.

Vriens et al. showed positive associations of sedentary behavior (represented by screen time) with body mass index, salivary miRNA-222 and miRNA-146a expression (33), while on the contrary, circulating plasma levels of miRNA-146a were up-regulated after acute physical activity in young endurance athletes (52). It is known that acute physical activity is associated with a transitory immunological/stress response, which in the long-term could be beneficial to the organism (53–55). In this context, miRNA-146a plays an essential role in the inflammatory signaling in different type of cells and might reflect the inflammatory state after prolonged aerobic physical activity (52). Thus, the increase of miRNA-146a after a single bout of physical activity might reflect the transitory stress/acute inflammatory response. However, high salivary miRNA-146a levels at resting conditions (i.e., not a transitory response to acute physical activity) could be interpreted as a biomarker of chronic inflammation, which might be related to higher body mass index, cardiovascular, and metabolic diseases. Importantly, several differences among studies must be considered for inferring biological implications, for example different tissues analyzed (saliva, plasma), populations (adolescents, young adults), and trained status (sedentary, endurance athletes, recreational or professional athletes).

On the other hand, Gopalan et al. did not report any differences on genes involved in the regulation of the immune and neurophysiological function (*IL-2* and *BDNF*) between “exercisers” (i.e., 24-weeks, 4 sessions/week 20–45 min + 15–30 min yoga) and “non-exercisers” children with HIV infection (29). To note, the duration of running and yoga was reported but the intensity of running (e.g., % of HRpeak or perceived exertion) was lacking. Another study by Lovinsky-Desir et al. showed that active children (at least 60 min of moderate-to-vigorous physical activity daily objectively measured by accelerometry) had lower methylation levels at *FOXP3* compared to non-active children (not met at least 60 min of moderate-to-vigorous physical activity daily), among those with higher air pollutant black carbon exposure (28). FOXP3 controls the differentiation and function of T regulatory cells, where increased *FOXP3* promoter methylation negatively associated with FOXP3 expression and linked to higher air pollution exposure (56). Furthermore, lung function outcomes such as the ratio between forced expiratory volume in 1 s (FEV1)/forced vital capacity (FVC) were negatively associated with *FOXP3* promoter methylation. These results suggest that urban children may obtain immunological/cardiorespiratory protection by an active lifestyle.

Wu et al. reported that substituting 30-min of vigorous physical activity for 30-min of sedentary behavior daily was associated with higher methylation levels at *HSD11B2* promoter in boys (40). *HSD11B* genes catalyze the interconversion of cortisol and corticosterone (46), and thereby vigorous acute physical activity might be associated with an increased transitory stress/immunological response (53–55). Thus, *HSD11B* could be involved in stress/immunological response to acute physical activity or related to vigorous physical activity levels. Besides, one study reported that higher PA levels (> 250 min/week) were related to a better inflammatory profile (up-regulation of *IL-10* and down-regulation of *TNF-*α) in children with type I diabetes (30). In summary, very few single genes have been analyzed and clearly bigger studies using the whole genome analysis approaches together with longitudinal studies are warranted.

### Acute Effects of Physical Activity

In our systematic review, three candidate-gene studies reported the acute effect of physical activity on gene expression. Kochanska-Dziurowicz et al. showed that an acute bout of physical activity on a cycle ergometer until voluntary exhaustion increased the beta-2 adrenergic receptor (*ADRB2*) gene expression in the whole-blood assessment of adolescent ice hockey players (39). ADRB2 is the main target of catecholamines such as noradrenaline (57) involved in the stress response (e.g., acute physical activity). A single bout of physical activity can increase the secretion of catecholamines, which in turn might decrease the production of pro-inflammatory markers such as IL-1β by immune cells (53). Thus, *ADRB2* gene up-regulation in the whole blood after a single bout of intense physical activity could be related to the anti-inflammatory effects of physical activity. Interestingly, the *ADRB2* gene was downregulated in lymphocytes of children with asthma (a disease characterized by a higher pro-inflammatory profile) compared to healthy controls (58). We hypothesize that *ADRB2* up-regulation after an intense acute bout of physical activity may induce partially immunological protection in children with asthma, which should be tested in future studies.

Kilian et al. used a cross-over design to test the impact of two different types of acute physical activity [e.g., High volume session (HVS) vs. High intensity interval training (HIIT)] on circulating miRNAs (miRNA-16, miRNA-21, and miRNA-126) and *VEGF* gene expression in healthy boys as competitive cyclists (35). *VEGF* gene and the abovementioned miRNAs are highly expressed in the endothelium cells and are involved in angiogenic processes such as the formation of new capillaries (35, 59, 60). MiRNA-16, miRNA-21, and miRNA-126 were up-regulated during and after HVS, while no changes were detected with the HIIT (4 × 4 min at 90–95% of peak power output with 3-min active recovery intervals at 45% of peak power output), suggesting that physical activity volume (duration) might play a role in regulating vascular circulating miRNA expression in children. On the other hand, Radom-Aizik et al. reported contrary results where miRNA-16 and miRNA-126 were down-regulated in neutrophils of healthy young adults after interval acute physical activity bouts (10 × 2 min at 76% VO2peak) (61). Different factors could explain partially the opposite findings between the two abovementioned studies (e.g., miRNA isolated from capillary blood from the earlobe *vs*. miRNA in circulating neutrophils, children vs. young adults, continuous vs. interval physical activity bouts). Future studies should analyze the impact of acute physical activity with different characteristics (e.g., volume, intensity, continuous or intermittent) on miRNA expression in children with different diseases and, if possible, performing high-throughput analyses (e.g., miRNA-sequencing).

Another study by Lu et al. reported up-regulation of *HSP70* after a single bout of physical activity (10 × 2 min at ∼75% of VO2peak with 1-min rest intervals), while the expression of candidate genes involved in the glucocorticoid receptor pathway (*GR*, *GR*β, *TGF*β*1*, and *TGF*β*2*) in PBMCs of asthmatics and healthy adolescents did not change (31). HSP70 protein can reduce the expression of pro-inflammatory cytokines such as TNF-α or IL-1β (62, 63), while physical activity can increase HSP70 levels in lymphocytes (63, 64). Besides, the coordinated action of HSP70 and HSP90 can regulate glucocorticoid receptor function (65). It is possible that other genes related to glucocorticoid function are also regulated by acute physical activity. This is a “limitation” of the single gene approach that could be overcome by using high-throughput analyses. Further research need to contrast or confirm whether the abovementioned genes could be altered in children with different clinical conditions such as obesity and implementing physical activity protocols with different characteristics.

In regards to high-throughput analyses, two studies reported the acute effects of physical activity (cycle ergometer test, 10 × 2 min bouts, ∼90% of HRpeak with 1-min rest intervals) on gene expression profile in PBMCs of healthy boys and girls at different pubertal stages using microarrays analysis (36, 37). The expression of 1,246 genes were altered following the acute physical activity bout in late-pubertal boys (37), while the expression level of 109 genes was found to be altered in early-pubertal boys (37). 13 gene pathways related to immune function and type I diabetes, among others were enriched (37). Contrary to boys, the difference in the number of genes their expression was altered following the same acute bout of physical activity was much smaller; 877 genes in late-pubertal girls (36) and 1,320 genes in early-pubertal girls (36). 622 genes overlapped between the groups. These genes enriched gene pathways involved in inflammation, stress, and apoptosis (36). These pioneering studies highlight the need to account for sex and pubertal stage when interpreting genomic data in response to acute bout of physical activity (36, 37), and the need to apply high-throughput approach to better understand the molecular mechanisms involved in the response to physical activity.

More research is needed to address the acute effects of physical activity on gene expression profile and its regulation in healthy children and children with clinical conditions (e.g., obesity, leukemia, anemia, cystic fibrosis) using high-throughput sequencing technologies (e.g., RNA-seq).

### Chronic Effects of Physical Activity

Only a few studies have investigated the chronic effects of physical activity on gene expression. Woo et al. reported the up-regulation of *SOD* and *GPX* in PBMCs of children with obesity and normal-weight after 12-weeks of physical activity intervention (32). These genes encode antioxidant enzymes (32), suggesting that long-term physical activity intervention (i.e., chronic effects) might improve the oxidative stress profile of these children. However, the characteristics of the physical activity intervention were unclear (i.e., intensity, frequency, type, among others) (32). Another study by Lu et al. reported that 8-week physical activity down-regulated the expression of *HSP70* in asthmatics and healthy adolescents (31), which can regulate glucocorticoid receptor function (65). Interestingly, acute physical activity altered the expression of *HSP70* in the opposite direction (i.e., up-regulated) indicating that acute and chronic physical activity may have a different impact on *HSP70* expression level.

A well-defined physical activity intervention based on HIIT (6-months, 2 sessions/week, 60 min/session at 80–95% HRmax with active breaks at 50–60% of HRmax) showed no changes in methylation levels at *RALBP1* in whole blood samples of adolescents with overweight/obesity (27). RALBP1 is involved in the pathogenesis of metabolic syndrome and obesity-associated inflammation (66, 67). Increased methylation at the *RALBP1* promoter region has been linked to decreased *RALBP1* gene expression in adipose tissue of healthy men after 6-months of physical activity intervention (one session of 1 h spinning and two sessions of 1 h aerobics per week, no more details were provided) (68). Similarly, a 12-week PA intervention based on indoor cycling (3 sessions/week, 60 min/session, intensity not specified) did not change the expression of the *ADRB2* gene in blood of children and adolescents with obesity (38). Interestingly, longitudinal changes (independent of PA intervention) on *ADRB2* gene expression were positively associated with fat loss (38). Otherwise, Zhao et al. showed that a 12-week PA intervention (5 sessions/week, 50 min/session at 60–70% of HR_*max*_.) decreased the expression of long non-coding RNA MALAT1 and increased the expression of miR-320a, which are related to endothelial function, in children with obesity (41). Heterogeneity among studies hampers comparisons and could partially explain the contradictory findings (e.g., different tissues, characteristics of physical activity interventions, age range of participants, and weight status).

It is evident that further studies should assess the chronic effects of physical activity attending to its different characteristics (i.e., type, intensity, volume, continuous vs. interval, etc.) on genes and molecular pathways in healthy children and children with different clinical conditions and training status. Besides, longitudinal randomized controlled trials (i.e., children randomly assigned into a physical activity or control group) are needed to characterize the molecular response to long-term physical activity interventions (i.e., chronic effects) in children and adolescents.

### Risk of Bias Assessment

The risk of bias assessment showed that physical activity should be measured using valid and reliable methods in future cross-sectional studies. Also, researchers should try to use objective methods such as accelerometry instead of self-report or questionnaires to quantify physical activity levels. Furthermore, regarding the acute effects of physical activity on gene expression, future studies should register nutritional intake, and physical activity performed 1–2 days before the exercise test to isolate the effect of physical activity performed on the test day from other confounding factors. Lastly, randomized controlled trials, including a physical activity group *vs.* control group, are needed to investigate the chronic effects of exercise on gene expression and epigenetic regulation in the pediatric population.

### Future Directions

Our systematic review highlights the small body of knowledge available to date on the genomic response to sedentary behavior and physical activity (i.e., acute and chronic effects) in children and adolescents. Most of the studies focused on candidate gene analyses, and only two studies performed high-throughput analyses to assess the acute effects of physical activity on gene expression profile in children. It is clear from the studies performed in adults and animals that sedentary behavior/physical activity alter the expression of thousands of gene in an orchestrated manner (15, 19, 22, 69). In order to move forward the knowledge of molecular effects of physical activity in children, randomized controlled trials (i.e., a group of children/adolescents performing physical activity *vs*. a control group following a usual lifestyle for weeks/months) applying the high-throughput omics techniques such as RNA/DNA sequencing together with epigenome analyses (genome-wide DNA methylation, acetylation, miRNA-seq) are warranted.

The main reason why research in the pediatric population is lagging behind other age groups is the limitation of obtaining study material for technical and ethical reasons. Interestingly, a recent study assessing 32 tissues in human concluded that the analysis of the whole blood gene expression predicts well the tissue-specific transcriptome profile (60% of the genes across 32 tissues), and especially accurate in the skeletal muscle (81% of the genes) and adipose tissue (75% of the genes) (70). This whole-blood transcriptome profile was a good predictor of different diseases such as type 2 diabetes or hypertension (70). Sedentary behavior and lack of physical activity are related to a higher cardiometabolic risk in the pediatric population and a higher risk of developing cardiovascular disease during adulthood (2, 8). Thus, future studies should investigate how sedentary lifestyles modify whole-blood transcriptome in children and adolescents, possibly contributing to the development of type 2 diabetes and hypertension later in life. These findings are promising and have the potential to enhance the field by increasing the number and the quality of studies exploring sedentary behavior/physical activity effects on genomic response in the pediatric population.

**FIGURE 3:**
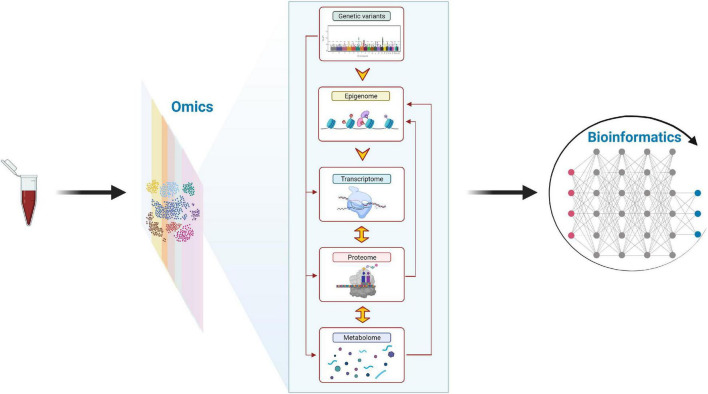
The complex integration of “omics” data (i.e., multi-omics analysis) might contribute to a better understanding of the molecular mechanisms underlying the health-related benefits of physical activity in children and adolescents. The human genome is essentially invariant and comprises more than 25,000 genes, which encode ∼100,000–200,000 transcripts and 1 million proteins, and a smaller number of metabolites (2,500–3,000) make up the human metabolome (71). The epigenome, which can be influenced by physical activity in adults (15), shows a low/moderate temporal variance and influences both transcriptome and proteome. The transcriptome can be affected by a single bout of physical activity (36, 37) in children and presents a high temporal variance and is translated into the proteome, influencing the metabolome in a tissue-specific manner. Figure modified from Altmäe et al. (72) with permission of the Publisher. This figure was created with BioRender.com.

Recently, Contrepois et al. investigated the effects of acute physical activity at molecular level performing multi-omics analyses in middle-aged adults (22). In the future, multi-omics analysis should be implemented to understand the link between changes on gene expression and epigenetics modifications at genome-wide level in response to physical activity (acute and chronic effects) in children and adolescents. Figure 3 illustrates the complexity and the potential of an integrated omics perspective. In this regard, the Molecular Transducers of Physical Activity Consortium (MoTrPAC) funded by National Institute of Health (NIH) Common Fund will address these gaps in 320 healthy children by characterizing the molecular response to physical activity (acute and chronic effects) performing multi-omics analyses (i.e., genomics, transcriptomics, epigenomics, metabolomics, proteomics) (69). The body of knowledge derived from MoTrPAC would be helpful to understand how physical activity is beneficial for health and how it can potentially be prescribed as a personalized medicine in children and adolescents. Further initiatives in this direction are needed to understand and unravel the complex effects of physical activity on health.

### Limitations and Strengths

This systematic review presents some limitations. First, a meta-analysis was not conducted due to the relatively small number of studies identified in this review and the heterogeneity reported between studies (i.e., different candidate genes selected, characteristics of the exposure, health status of the participants, and study designs). Second, our review did not report dissertations, conference proceedings, and trial registries (i.e., gray literature). Third, articles using different languages than English or Spanish were not considered for eligibility. Otherwise, our systematic review has several strengths. First, PRISMA guidelines were followed to ensure the scientific rigor for elaborating this systematic review. Second, research questions and inclusion/exclusion criteria were thought out and elaborated on before conducting this systematic review, as shown in the registration in the PROSPERO database. Third, three different electronic databases (PubMed, Web of Science, and Scopus) were used to detect articles, and the search strategies terms for each database are reported in Supplementary Table 1). Fourth, we reported the risk of bias assessment of articles included in this review, which was performed using specific valid tools for cross-sectional studies, non-randomized controlled trials [Joanna Briggs Institute Critical Appraisal Tool for Systematic Reviews (24)], and acute effects of physical activity studies [modified version of the Downs and Black checklist (25)].

## Conclusion

This review highlights how scarce is our knowledge on the molecular effects of sedentary behavior and physical activity on pediatric population. The few studies in the field suggest that sedentary behavior and physical activity are associated with miRNA expression and DNA methylation, where the most relevant candidate genes in the cross-sectional studies were: *FOXP3*, *HSD11B2*, *IL-10, TNF-*α, and non-coding RNAs miRNA-222, and miRNA-146a. The acute or chronic effects of physical activity regulated the expression of genes *ADRB2, HSP70, SOX*, *GPX*, and non-coding RNAs such as miRNA-16, miRNA-126, miR-320a, and long non-coding RNA MALAT1. Transcriptomic analyses identified gene pathways involved in inflammation, apoptosis or diabetes among others, that are influenced by acute physical activity in boys and girls. In conclusion, very few genes and genetic regions have been studied, and the field is yawing for studies on bigger cohorts, longitudinal studies and randomized controlled trials together with high-throughput multi-omics analyses.

## Data Availability Statement

The original contributions presented in this study are included in the article/Supplementary Material, further inquiries can be directed to the corresponding author/s.

## Author Contributions

AP-F conceptualized, designed, and wrote the manuscript. IP-P participated in all systematic review phases (except for writing), elaborated the figure summary, and reviewed the initial manuscript draft for important intellectual content. PM-G participated in elaborating the search strategy, contributed to methodological aspects such as the risk of bias assessment, and reviewed the initial manuscript draft for important intellectual content. SR-A reviewed the manuscript for important intellectual content. FO and SA conceptualized and designed the review, supervised all the article processes, and reviewed the manuscript for important intellectual content. All authors contributed to the article and approved the submitted version.

## Conflict of Interest

The author SA is collaborating with the Competence Centre on Health Technologies (Estonia) and is not employed by the entity. The remaining authors declare that the research was conducted in the absence of any commercial or financial relationships that could be construed as a potential conflict of interest.

## Publisher’s Note

All claims expressed in this article are solely those of the authors and do not necessarily represent those of their affiliated organizations, or those of the publisher, the editors and the reviewers. Any product that may be evaluated in this article, or claim that may be made by its manufacturer, is not guaranteed or endorsed by the publisher.

## Footnotes

1
https://www.covidence.org/
