# Corrigendum: Transcriptional and epigenetic response to sedentary behavior and physical activity in children and adolescents: A systematic review

**DOI:** 10.3389/fped.2022.993123

**Published:** 2022-08-10

**Authors:** Abel Plaza-Florido, Inmaculada Pérez-Prieto, Pablo Molina-Garcia, Shlomit Radom-Aizik, Francisco B. Ortega, Signe Altmäe

**Affiliations:** ^1^Department of Physical and Sports Education, Faculty of Sport Sciences, PROFITH “PROmoting FITness and Health Through Physical Activity” Research Group, Sport and Health University Research Institute (iMUDS), University of Granada, Granada, Spain; ^2^Department of Biochemistry and Molecular Biology, Faculty of Sciences, University of Granada, Granada, Spain; ^3^Instituto de Investigación Biosanitaria (ibs.GRANADA), Granada, Spain; ^4^Physical Medicine and Rehabilitation Service, Virgen de las Nieves University Hospital, Granada, Spain; ^5^Pediatric Exercise and Genomics Research Center, UC Irvine School of Medicine, Irvine, CA, United States; ^6^Faculty of Sport and Health Sciences, University of Jyväskylä, Jyväskylä, Finland; ^7^Department of Biosciences and Nutrition, Karolinska Institutet, Huddinge, Sweden; ^8^Division of Obstetrics and Gynecology, CLINTEC, Karolinska Institutet, Stockholm, Sweden; ^9^Competence Centre on Health Technologies, Tartu, Estonia

**Keywords:** exercise, methylation, omics, physical fitness, RNA-seq, epigenomics

In the original article, we neglected to include the affiliation number 3 for the author Pablo Molina-Garcia. The affiliation added is “^3^ Instituto de Investigación Biosanitaria (ibs.GRANADA), Granada, Spain.”

In the original article, the reference “37. Radom-Aizik S, Zaldivar F, Leu SY, Cooper DM. Brief bout of exercise alters gene expression in peripheral blood mononuclear cells of early- and late-pubertal males. *Pediatr Res*. (2009) 65:447–52. doi: 10.1203/PDR.0b013e3181993473” was missing. The reference list has been updated.

In the original article, the correct reference number “37. Radom-Aizik S, Zaldivar F, Leu SY, Cooper DM. Brief bout of exercise alters gene expression in peripheral blood mononuclear cells of early- and late-pubertal males. *Pediatr Res*. (2009) 65:447–52. doi: 10.1203/PDR.0b013e3181993473” was not cited in the article.

The citation has now been inserted in the **Results** section, Paragraph one and Paragraph three, and the **Discussion** section, Paragraph one and Paragraph nine. These paragraphs appear below.

In the original article, there was an error in Table 1. “Histone acetylation” and “Microarray” are different terms and were combined in the table row. “qPCR” and “tanscriptome” are different terms and were combined in the table row. The corrected Table 1 appears below.

**Table 1:** Definition of the main molecular biology-related terms used in this systematic review.

**Term**	**Definition**
mRNA	Messenger RNA (mRNA) carries the genetic information from nucleus to ribosomes necessary to synthesize proteins. Gene expression analysis is based on analysing mRNA molecules.
Epigenetics	Epigenetic modifications (i.e., DNA methylation, histone acetylation) that act on DNA structure. These mechanisms can activate or repress transcription (i.e., gene expression). miRNA is also considered a form of epigenetic regulation, see description below.
CpG site	DNA region prone to methylation where a cytosine nucleotide is followed by a guanine nucleotide linked by a phosphate group.
DNA methylation	One of the most studied epigenetic modifications that consists in adding a methyl group to C nucleotide in DNA.
Histone acetylation	Epigenetic modification that involves the addition of an acetyl group to the histone proteins.
Microarray	Microarray is a technology that detects the expression levels of thousands of genes at the same time. Briefly, thousands of genetic sequences are located on a chip, and based on the complementary sequences of the transcripts in a biological sample the hybridization takes place, allowing the detection of gene expression levels.
miRNA	Non-coding micro RNA (miRNA) molecule that is small in length, 18–24 pair of bases. These small RNA molecules are able to regulate gene expression by influencing the half-life of the mRNA or it's availability for translation.
omics	Refers to analyses of entire set of molecules such as proteins (i.e., proteomics), metabolites (i.e., metabolomics), DNA sequence variants (i.e., genomics), mRNA expression (i.e., transcriptomics), or DNA methylation profile (i.e., epigenomics) within the sample.
RNA-seq	RNA sequencing technique to quantity the gene expression profile (i.e., transcriptome) in a biological sample.
qPCR	Laboratory technique based on polymerase chain reaction (PCR), which is widely used in molecular biology to amplify a specific nucleic acid sequence and obtain millions to billions of copies. This technique is able to quantify gene expression levels.
Transcriptome	Analysis of transcripts (typically mRNA molecules) in order to assess the gene expression levels. Both microarray and RNA-seq approaches are used. The difference between these methods is that in the array a set of possible genes is defined by the set of probes that are present, while RNA-seq allows detection of known and unknown genes.

In the original article, there was an error in Table 2, the reference number 37 was indicated for different manuscripts as follows “Radom-Aizik et al. (37)” and “de Souza e Silva et al. (37).” The correct Table 2 appears below.

**Table 2:** Summary of study characteristics of articles included in this review.

**Sedentary behavior and physical activity: cross-sectional evidence**						
**References**	**Study design**	**Target population [Sample size (** ***N*** **)]; Sex (boys %); Age (SD or range in years); Ethnicity/Race (%)**	**Characteristics of the exposure (SB, PA) or PA intervention**	**Tissue**	**Dependent outcome and analytical method**	**Main findings**
Wu et al. (34)	Cross-sectional	Group 1: Children with obesity (*N* = 59); Boys + Girls (45.8%); 13.8 ± 3.0 y; Chinese (100%) Group 2: Normal-weight children (*N* = 39); Boys + Girls (61.5%); 10.3 ± 1.1 y; Chinese (100%)	SB and PA across 6 months (questionnaire completed by parents or guardians)	Leukocytes	DNA methylation at *FAIM2* promoter (Sequenom MassARRAY platform)	Differentially methylation levels at *FAIM2* promoter between obese and normal-weight children according to SB and PA levels. Results were not significant after multiple hypothesis testing corrections
Lovinsky-Desir et al. (28)	Cross-sectional	Group 1: Active children (*N* = 77); Boys + Girls (45%); 12.2 y (9.2–14.0 y); Hispanic (60%), African American (40%) Group 2: Non-active children (*N* = 58); Boys + Girls (55%); 12.7 y (10.5–14.0 y); Hispanic (72%), African American (28%)	PA across 6 days (accelerometer on the non-dominant wrist)	Buccal swabs (squamous epithelial cells)	DNA methylation at *FOXP3* promoter (pyrosequencing) and gene expression	Active children had lower *FOXP3* promoter methylation compared to Non-active children exposed to high air pollutant black carbon concentrations. No significant association was reported between FOXP3 promoter methylation and gene expression
Vriens et al. (33)	Cross-sectional	Children with normal-weight 70%, overweight 12.5%, and underweight 17.5% (*N* = 80); Boys + Girls (46.3%); 10.44 ± 0.97 y; Caucasian (91.3%)	SB and PA across ~2 years (out-of-school sport activities and screen time use questionnaires filled out by the parents)	Extracellular fraction of saliva	Expression levels of miRNA-222 and miRNA-146a (qPCR)	SB, represented by screen time use, was positively associated with miRNA-222 and miRNA-146a levels. PA was not significantly associated with either miRNA-222 or miRNA-146a
Wu et al. (40)	Cross-sectional	Adolescents (*N* = 369); Boys + Girls (47.2%); 14.22 ± 1.99 y for boys/13.95 ± 2.04 y for girls; Mexican (100 %)	SB and PA across 7 days (accelerometer on the non-dominant wrist)	Leukocytes	DNA methylation at *PPARA, H19, LINE-1*, and *HSD11B2* (pyrosequencing)	Substituting 30-min of vigorous PA for 30-min of SB daily was associated with higher methylation at *HSD11B2* promoter in boys
**References**	**Study design**	**Target population [Sample size (** ***N*** **)]; Sex (boys %); Age (SD or range in years); Ethnicity/Race (%)**	**Characteristics of the exposure (SB, PA) or PA intervention**	**Tissue**	**Dependent outcome and analytical method**	**Main findings**
Gopalan et al. (29)^a^	Cross-sectional	Group 1: Exercisers (*N* = 20); Boys + Girls with HIV infection (75%); 10.5 y; Indian (100%) Group 2: Non-exercisers (*N* = 20); Boys + Girls with HIV infection (44.4%); 12.5 y; Indian (100%)	Children who practiced 20–45 min/day, 4 times per week from year 0 to year 2 were categorized as “exercisers” (physical activity questionnaire suited for Indian children)	PBMC	*IL-2* and *BDNF* gene expression (qPCR)	The gene expression of *IL-2* and *BDNF* was not significantly different between exercisers and non-exercisers groups
Dos Santos Haber et al. (30)	Cross-sectional	Children and adolescents (*N* = 108) divided into 4 groups (type I diabetes with ketoacidosis; decompensated type I diabetes; Compensated type I diabetes and healthy control); Boys + girls (NR); 10-18 years old; NR	Frequency and duration of PA activities recorded during the last 3 months by questionnaires. Children were classified as low active (<150 min/week), active (150–250 min/week), and very active (>250min/week)	Blood samples	*IL-10* and *TNF-α* (qPCR)	A higher PA level (very active compared to active and control groups) was associated with increased IL-10 and decreased TNF-α expression in children with type I diabetes/ketoacidosis and decompensated type I diabetes
**Acute effects of physical activity**						
Radom-Aizik et al. (37)	Within-subjects experiment	Group 1: Early-pubertal boys (*N* = 10); Boys; 10.5 + 0.4 y; NR Group 2: Late-pubertal boys (*N* = 10); Boys; 17.4 + 0.4 y; NR	Cycle ergometer test, 10 ×2 min bouts, the work rate was individualized for each boy (~90% of HR*_*peak*_*) with 1-min rest intervals	PBMC	Microarray gene expression (Affymetrix U133+2 arrays)	A single bout of PA induced changes in PBMC gene expression in both groups, particularly 1,246 genes (517 up, 729 down) in late-pubertal boys and 109 (79 up, 30 down) in early pubertal boys. 13 gene pathways involved in immune function and type I diabetes, were altered by acute PA in both early- and late-pubertal boys
**References**	**Study design**	**Target population [Sample size (** ***N*** **)]; Sex (boys %); Age (SD or range in years); Ethnicity/Race (%)**	**Characteristics of the exposure (SB, PA) or PA intervention**	**Tissue**	**Dependent outcome and analytical method**	**Main findings**
Radom-Aizik et al. (36)	Within-subjects experiment	Group 1: Early-pubertal girls (*N* = 10); Girls; 10.0 + 0.3 y; NR Group 2: Late-pubertal girls (*N* = 10); Girls; 16.1 + 0.4y; NR	Cycle ergometer test, 10 ×2 min bouts, the work rate was individualized for each girl (~90% of HR*_*peak*_*) with 1-min rest intervals	PBMC	Microarray gene Expression (Affymetrix U133 + 2 arrays)	A single bout of PA induced changes in PBMC gene expression in both groups, particularly, 877 genes (611 up, 266 down) in late-pubertal girls and 1,320 (829 up, 491 down) in early-pubertal girls. 5 gene pathways related to inflammation, stress, and apoptosis, were altered by acute PA in both early- and late-pubertal girls
Kochanska-Dziurowicz et al. (39)	Within-subjects experiment	Youth ice hockey players (*N* = 19); Boys; 17.1 ± 0.5 y; Polish (100%)	Cycle ergometer test until voluntary exhaustion (starting with 1.0 W∙kg^−1^ load and increasing the intensity by 0.5 W∙kg^−1^ each 3 min)	PBMC	*ADRB2* and *ACTB* gene expression (qPCR)	ADRB2 and ACTB (internal control) gene expression increased in 74% of players after the PA test
Kilian et al. (35)	Cross-over experiment	Competitive young cyclists (*N* = 12); Boys; 14.4 ± 0.8 y; NR	Session 1: HIIT, 4 ×4 min at 90-95% PPO with 3-min active recovery intervals at 45% PPO Session 2: HVT, 90 min at 60% PPO	Capillary blood samples	Expression levels of miRNA-16, miRNA-21, miRNA-126, and VEGF mRNA (qPCR)	HVT significantly increased miRNA-16 and miRNA-126 during and after the PA test, whereas HIIT showed no significant influence on the miRNAs. VEGF gene expression significantly increased during and after HIIT and HVT
**References**	**Study design**	**Target population [Sample size (** ***N*** **)]; Sex (boys %); Age (SD or range in years); Ethnicity/Race (%)**	**Characteristics of the exposure (SB, PA) or PA intervention**	**Tissue**	**Dependent outcome and analytical method**	**Main findings**
Lu et al. (31)^b^	Within-subjects experiment	Group 1: Asthmatics adolescents (*N* = 12); Boys + Girls (33.3%); 15.7 y (14.0–17.0 y); White (50%), Asian (42%), more than one ethnicity (1%) Group 2: Healthy adolescents (*N* = 14); Boys + Girls (57.1%); 15.0 y (14.0–17.0 y); White (71%), Asian (21%), more than one ethnicity (7%)	Acute effects of PA: Cycle ergometer test, 10 ×2 min at ~75% of VO*_2*peak*_* with 1-min rest intervals Chronic effects of PA: 8-weeks, 3 days/week (1 h-session)	PBMC	*GR (NR3C1), GR*β, *HSP70, TGF*β*1*, and *TGF*β*2* gene expression (qPCR)	No effect on PBMC gene expression of *NR3C1, GR*β, *TGF*β*1*, and *TGF*β*2* in both healthy and asthmatic adolescents. In addition, HSP70 gene expression was increased after acute PA while was decreased after chronic PA intervention
**Chronic effects of physical activity**						
Woo et al. (32)^c^	Non-randomized controlled trial	Group 1: Children with overweight (*N* = 20); Boys; 11.30 ± 1.17 y; Korean (100%) Group 2: Normal-weight children (*N* = 19); Boys; 11.32 ± 1.06 y; Korean (100 %)	12-weeks PA intervention. The characteristics of the PA intervention were unclear (i.e., intensity, frequency, among others)	PBMC	*SOD* and *GPX* gene expression (qPCR)	*SOD* and *GPX* gene expression was up-regulated after 12-weeks of PA in both groups. In addition, *SOD* and *GPX* gene expression was up-regulated after 24-weeks of PA in children with overweight
Blüher et al. (27)	Non-randomized controlled trial	Adolescents with overweight/obesity (*N* = 28); Boys + Girls (46.5%); 15.5 ± 1.4 y; NR	HIIT, 6-months, 2 sessions/week, 60 min/session at 80–95% HR*_*max*_* with active breaks at 50–60% of HR*_*max*_*	Blood samples	DNA methylation at *RALBP1* (pyrosequencing)	No significant changes in levels of methylation at *RALBP1* were observed after 6-months of PA intervention in children with overweight/obesity
Zhao et al. (41)	Non-randomized controlled trial	Children and adolescents with obesity (PA intervention group *N* = 40; control group *N* = 20); Boys + Girls (68.3%); 8–16 y; NR	12-weeks PA intervention. Frequency of 5 sessions/week, 50 min each session, intensity 60–70% of HR*_*max*_*	Blood samples	Long non-coding RNA MALAT1 and miR-320a expression (qPCR)	PA intervention decreased MALAT1 and increased miR-320a expression
De Souza E Silva et al. (38)	Non-randomized controlled trial	Children and adolescents with overweight/obesity (PA intervention group *N* = 17; control group *N* = 18); Boys + Girls (53.0%); 10–16 y; Euro-Brazilian (self-reported)	12-weeks PA intervention (indoor cycling), 3 sessions/week (60 min/session)	Blood samples	*ADRB2* gene expression (qPCR)	No significant changes in levels of *ADRB2* expression were reported after 12-weeks of PA intervention in children with overweight/obesity

In the original article, there was an error in the legend of Figure 3, the reference number 37 was missing. The correct legend appears below.

In the original article, we neglected to include the funders The Estonian Research Council (grant PRG1076), and the European Commission and Enterprise Estonia (grant EU48695). The correct Funding statement appears below.

In the original article, the **Conflict of Interest** statement was incomplete. Author Signe Altmäe was collaborating with Competence Centre on Health Technologies, Estonia. The corrected statement appears below.

**Results**, Paragraph one

“PRISMA checklist 2020 shows the appropriateness of the methods performed in our systematic review (**Supplementary Tables 2, 3**). Figure 1 illustrates the PRISMA 2020 flow diagram for the selection process of the studies: a total of 1,473 articles were included from the three databases, and after removing the duplicates and non-eligible studies, 15 articles remained eligible for this review (6 cross-sectional articles, 5 studies reported the acute effects of physical activity, and 5 articles showed the chronic effects of physical activity). The sample size ranged from 12 to 369 participants (27–41). The age of participants ranged from 9 to 18 years old (27–41). Thirteen studies used blood samples (27, 29–32, 34–41) while 2 saliva (33) and buccal swabs (28) respectively. Regarding disease, four studies included children with obesity (27, 34, 38, 41) and 1 study children with HIV infection (29). Concerning countries/regions, 4 studies were performed in the United States of America (28, 31, 36, 37), 2 in Brazil (30,38), 4 in Europe (27, 33, 35, 39), 3 in Asia (32, 34, 41), 1 in Mexico (40), and 1 in India (29). All the relevant information extracted from each article is presented in Table 2. In addition, a graphical summary of the mains results is presented in Figure 2. Specific genes and related pathways found in the studies are interpreted and discussed in the context of existing knowledge in the Discussion section.”

**Results**, Paragraph three

“Five out of the twelve articles presented in Table 2 reported significant effects of acute bout of physical activity on gene expression (31, 35–37, 39). Among the five studies, three reported the effects of acute bout of physical activity using candidate gene analyses (i.e., mRNA or miRNA expression) (31, 35, 39), while two studies performed high-throughput transcriptomics analyses using microarrays (36, 37). Four studies used circulating peripheral blood mononuclear cells (PBMCs) to quantify gene expression (31, 36, 37, 39), while one study used capillary blood samples from the earlobe (35).”

**Discussion**, Paragraph one

“This study aimed to provide current knowledge on the effect of sedentary behavior and physical activity on gene expression and epigenetic mechanisms in the pediatric population. The main findings and gaps identified by this systematic review in children and adolescents were: (1) there is very limited information of the molecular mechanisms of sedentary behavior and/or physical activity on gene expression and its regulation in pediatric population; (2) most of the studies showed that sedentary behavior and physical activity (acute and chronic effects) alter gene and MicroRNA expression, and DNA methylation of candidate genes related to obesity, asthma, immune function, and cardiovascular disease; (3) the studies are hardly comparable due to different candidate genes selected, characteristics of the exposure, health and training status of the participants, and study designs; (4) only two studies performed high-throughput transcriptomics analyses and detected thousands of genes differentially altered by acute bout of physical activity in boys and girls at different pubertal stages (36, 37). The relatively small number of studies, the heterogeneity in the methodology, different study designs, and most of the studies were performed in Europe and/or the United States of America (8/15) limit the extrapolation of our findings to the general pediatric population. Studies using high-throughput techniques (i.e., sequencing) and longitudinal study approach and/or randomized controlled trials on bigger cohorts are lacking in children and adolescents.”

**Discussion**, Paragraph nine

“In regards to high-throughput analyses, two studies reported the acute effects of physical activity (cycle ergometer test, 10 ×2 min bouts, ~90% of HRpeak with 1-min rest intervals) on gene expression profile in PBMCs of healthy boys and girls at different pubertal stages using microarrays analysis (36, 37). The expression of 1,246 genes were altered following the acute physical activity bout in late-pubertal boys (37), while the expression level of 109 genes was found to be altered in early-pubertal boys (37). 13 gene pathways related to immune function and type I diabetes, among others were enriched (37). Contrary to boys, the difference in the number of genes their expression was altered following the same acute bout of physical activity was much smaller; 877 genes in late-pubertal girls (36) and 1,320 genes in early-pubertal girls (36). 622 genes overlapped between the groups. These genes enriched gene pathways involved in inflammation, stress, and apoptosis (36). These pioneering studies highlight the need to account for sex and pubertal stage when interpreting genomic data in response to acute bout of physical activity (36, 37), and the need to apply high-throughput approach to better understand the molecular mechanisms involved in the response to physical activity.”

**Figure 3**. The complex integration of “omics” data (i.e., multi-omics analysis) might contribute to a better understanding of the molecular mechanisms underlying the health-related benefits of physical activity in children and adolescents. The human genome is essentially invariant and comprises more than 25,000 genes, which encode ~100,000–200,000 transcripts and 1 million proteins, and a smaller number of metabolites (2,500–3,000) make up the human metabolome (71). The epigenome, which can be influenced by physical activity in adults (15), shows a low/moderate temporal variance and influences both transcriptome and proteome. The transcriptome can be affected by a single bout of physical activity (36, 37) in children and presents a high temporal variance and is translated into the proteome, influencing the metabolome in a tissue-specific manner. Figure modified from Altmäe et al. (72) with permission of the Publisher. This figure was created with BioRender.com.

## Funding

The project was funded by the Spanish Ministry of Economy and Competitiveness (Reference DEP2013-47540, DEP2016-79512-R, and DEP2017-91544-EXP); the European Regional Development Fund (FEDER): grants RYC-2016-21199 and ENDORE SAF2017-87526-R. AP-F and IP-P were supported by the Spanish Ministry of Education, Culture and Sport (FPU 16/02760; FPU19/05561). SA was supported by NIH UO1 TR002004 and PERC Systems Biology Fund. This research was partly funded by Huawei Technologies, Finland. Additional support was obtained from the EXERNET Research Network on Exercise and Health (DEP2005- 00046/ACTI; 09/UPB/19; 45/UPB/20; 27/UPB/21); Alicia Koplowitz Foundation. This study has been partially funded by the University of Granada, Plan Propio de Investigación 2016, Excellence actions: Units of Excellence; Unit of Excellence on Exercise and Health (UCEES), and by the Junta de Andalucía, Consejería de Conocimiento, Investigación y Universidades and European Regional Development Fund (ERDF), ref. SOMM17/6107/UGR. Additional funding was obtained from the Andalusian Operational Program supported with European Regional Development Funds (FEDER) projects ref: B-CTS-355,UGR18, B-CTS-500-UGR18 and A-CTS-614-UGR20; and the Junta de Andalucía (PAIDI P20_00158). The Estonian Research Council (grant PRG1076); the European Commission and Enterprise Estonia (grant EU48695).

## Conflict of interest

The author SA is collaborating with the Competence Centre on Health Technologies (Estonia) and is not employed by the entity. The remaining authors declare that the research was conducted in the absence of any commercial or financial relationships that could be construed as a potential conflict of interest. The authors apologize for these errors and state that this does not change the scientific conclusions of the article in any way. The original article has been updated.

## Publisher's note

All claims expressed in this article are solely those of the authors and do not necessarily represent those of their affiliated organizations, or those of the publisher, the editors and the reviewers. Any product that may be evaluated in this article, or claim that may be made by its manufacturer, is not guaranteed or endorsed by the publisher.

