# Supplementary material for: Transcriptional and Epigenetic Response to Sedentary Behavior and Physical Activity in Children and Adolescents: A Systematic Review
Source: Front Pediatr. 2022 Jun 24;10:917152. doi: 10.3389/fped.2022.917152 (PMC9263076; doi:10.3389/fped.2022.917152)
Supplement: Supplementary file 1 [file Table_1.DOCX]

**Table S1.** Search terms used in PubMed, Web of Science, and Scopus databases

| **PubMed (N papers = 339)** |
| --- |
| **Population**  Title/Abstract(child* OR adolescen* OR youth* OR teenager* OR boy* OR girl*) |
| **Exposure**  Title/Abstract("screen time" OR "screen times" OR "sedentary behaviour" OR "Sedentary Behaviors" OR "Sedentary Lifestyle" OR "Sedentary Lifestyles" OR "Physical Inactivity" OR exercise* OR "Physical Activity" OR "Physical Activities" OR "sitting") |
| **Outcomes**  Title/Abstract(transcriptom* OR epigenom* OR "omics" OR "gene expression" OR "Expression, Gene" OR "Expressions, Gene" OR "Gene Expressions" OR "histones" OR Acetylation* OR microarray* OR "RNA-Seq" OR "Whole Transcriptome Shotgun Sequencing" OR "Whole Transcriptome Sequencing" OR "RNA" OR "ribonucleic Acid" OR Methylation* OR "MicroRNA" OR "miRNAs" OR "Micro RNA" OR "miRNA" OR "stRNA" OR epigenetic* OR "sequencing") |
| **Web of Science (N papers = 730)** |
| **Population**  TS=(child* OR adolescen* OR youth* OR teenager* OR boy* OR girl*) |
| **Exposure**  TS=("screen time" OR "screen times" OR "sedentary behaviour" OR "Sedentary Behaviors" OR "Sedentary Lifestyle" OR "Sedentary Lifestyles" OR "Physical Inactivity" OR exercise* OR "Physical Activity" OR "Physical Activities" OR "sitting") |
| **Outcomes**  TS=(transcriptom* OR epigenom* OR "omics" OR "gene expression" OR "Expression, Gene" OR "Expressions, Gene" OR "Gene Expressions" OR "histones" OR Acetylation* OR microarray* OR "RNA-Seq" OR "Whole Transcriptome Shotgun Sequencing" OR "Whole Transcriptome Sequencing" OR "RNA" OR "ribonucleic Acid" OR Methylation* OR "MicroRNA" OR "miRNAs" OR "Micro RNA" OR "miRNA" OR "stRNA" OR epigenetic* OR "sequencing") |
| **Scopus (N papers = 404)** |
| **Population**  TITLE-ABS (child* OR adolescen* OR youth* OR teenager* OR boy* OR girl*) |
| **Exposure**  TITLE-ABS ("screen time" OR "screen times" OR "sedentary behaviour" OR "Sedentary Behaviors" OR "Sedentary Lifestyle" OR "Sedentary Lifestyles" OR "Physical Inactivity" OR exercise* OR "Physical Activity" OR "Physical Activities" OR "sitting") |
| **Outcomes**  TITLE-ABS (transcriptom* OR epigenom* OR "omics" OR "gene expression" OR "Expression, Gene" OR "Expressions, Gene" OR "Gene Expressions" OR "histones" OR acetylation* OR microarray* OR "RNA-Seq" OR "Whole Transcriptome Shotgun Sequencing" OR "Whole Transcriptome Sequencing" OR "RNA" OR "ribonucleic Acid" OR methylation* OR "MicroRNA" OR "miRNAs" OR "Micro RNA" OR "miRNA" OR "stRNA" OR epigenetic* OR "sequencing") |
