# Supplementary material for: Transcriptional and Epigenetic Response to Sedentary Behavior and Physical Activity in Children and Adolescents: A Systematic Review
Source: Front Pediatr. 2022 Jun 24;10:917152. doi: 10.3389/fped.2022.917152 (PMC9263076; doi:10.3389/fped.2022.917152)
Supplement: Supplementary file 2 [file Table_2.docx]

**Table S2**. PRISMA main checklist 2020

| **Topic** | **No.** | **Item** | **Location where item is reported** |
| --- | --- | --- | --- |
| **TITLE** |  |  |  |
| **Title** | 1 | Identify the report as a systematic review. | Yes, page 1 |
| **ABSTRACT** |  |  |  |
| **Abstract** | 2 | See the PRISMA 2020 for Abstracts checklist | See Table S3 |
| **INTRODUCTION** |  |  |  |
| **Rationale** | 3 | Describe the rationale for the review in the context of existing knowledge. | Yes. Page 2 |
| **Objectives** | 4 | Provide an explicit statement of the objective(s) or question(s) the review addresses. | Yes. Page 2 |
| **METHODS** |  |  |  |
| **Eligibility criteria** | 5 | Specify the inclusion and exclusion criteria for the review and how studies were grouped for the syntheses. | Yes. Page 2 |
| **Information sources** | 6 | Specify all databases, registers, websites, organisations, reference lists and other sources searched or consulted to identify studies. Specify the date when each source was last searched or consulted. | Yes. Page 2 |
| **Search strategy** | 7 | Present the full search strategies for all databases, registers and websites, including any filters and limits used. | Yes, presented in supplementary Table 1 and reflected in the page 2 |
| **Selection process** | 8 | Specify the methods used to decide whether a study met the inclusion criteria of the review, including how many reviewers screened each record and each report retrieved, whether they worked independently, and if applicable, details of automation tools used in the process. | Yes. Page 2 |
| **Data collection process** | 9 | Specify the methods used to collect data from reports, including how many reviewers collected data from each report, whether they worked independently, any processes for obtaining or confirming data from study investigators, and if applicable, details of automation tools used in the process. | Yes. Pages 2 and 3 |
| **Data items** | 10a | List and define all outcomes for which data were sought. Specify whether all results that were compatible with each outcome domain in each study were sought (e.g. for all measures, time points, analyses), and if not, the methods used to decide which results to collect. | Yes. Pages 2 and 3 |
|  | 10b | List and define all other variables for which data were sought (e.g. participant and intervention characteristics, funding sources). Describe any assumptions made about any missing or unclear information. | Yes. Pages 3-4 |
| **Study risk of bias assessment** | 11 | Specify the methods used to assess risk of bias in the included studies, including details of the tool(s) used, how many reviewers assessed each study and whether they worked independently, and if applicable, details of automation tools used in the process. | Yes. Page 3 |
| **Effect measures** | 12 | Specify for each outcome the effect measure(s) (e.g. risk ratio, mean difference) used in the synthesis or presentation of results. | Not applicable |
| **Synthesis methods** | 13a | Describe the processes used to decide which studies were eligible for each synthesis (e.g. tabulating the study intervention characteristics and comparing against the planned groups for each synthesis (item 5)). | Yes. Page 4 reflect the study selection and data extraction |
|  | 13b | Describe any methods required to prepare the data for presentation or synthesis, such as handling of missing summary statistics, or data conversions. | Yes. Pages 3 and 4 reflect the study selection and data extraction |
|  | 13c | Describe any methods used to tabulate or visually display results of individual studies and syntheses. | Yes. Table 2 in the main manuscript and Figure 2 |
|  | 13d | Describe any methods used to synthesize results and provide a rationale for the choice(s). If meta-analysis was performed, describe the model(s), method(s) to identify the presence and extent of statistical heterogeneity, and software package(s) used. | Not applicable |
|  | 13e | Describe any methods used to explore possible causes of heterogeneity among study results (e.g. subgroup analysis, meta-regression). | Not applicable. The heterogeneity is due to different candidate genes selected and study designs because we included cross-sectional, acute physical activity and chornic exercise effects |
|  | 13f | Describe any sensitivity analyses conducted to assess robustness of the synthesized results. | Not applicable |
| **Reporting bias assessment** | 14 | Describe any methods used to assess risk of bias due to missing results in a synthesis (arising from reporting biases). | Yes. Page 3 |
| **Certainty assessment** | 15 | Describe any methods used to assess certainty (or confidence) in the body of evidence for an outcome. | Risk of bias might reflect validity and reliability of the outcomes in the 15 studies included in this review article. Supplementary tables 4,5, and 6 |
| **RESULTS** |  |  |  |
| **Study selection** | 16a | Describe the results of the search and selection process, from the number of records identified in the search to the number of studies included in the review, ideally using a flow diagram. | Yes, pages 3 and 4 |
|  | 16b | Cite studies that might appear to meet the inclusion criteria, but which were excluded, and explain why they were excluded. | The PRISMA diagram (Figure 1 in the manuscript) shows the reasons of exclusions and the number of studies excluded for each specific reason |
| **Study characteristics** | 17 | Cite each included study and present its characteristics. | Yes, Table 2 |
| **Risk of bias in studies** | 18 | Present assessments of risk of bias for each included study. | Yes, page 4 and 6 and supplementary Tables 4, 5, and 6 |
| **Results of individual studies** | 19 | For all outcomes, present, for each study: (a) summary statistics for each group (where appropriate) and (b) an effect estimate and its precision (e.g. confidence/credible interval), ideally using structured tables or plots. | Not applicable, summary of the study characteristics and results is presented in Table 2 |
| **Results of syntheses** | 20a | For each synthesis, briefly summarise the characteristics and risk of bias among contributing studies. | Yes, risk bias assessment page 4 and supplementary Tables 4, 5, and 6 |
|  | 20b | Present results of all statistical syntheses conducted. If meta-analysis was done, present for each the summary estimate and its precision (e.g. confidence/credible interval) and measures of statistical heterogeneity. If comparing groups, describe the direction of the effect. | Not applicable |
|  | 20c | Present results of all investigations of possible causes of heterogeneity among study results. | Not applicable. The heterogeneity is due to the limited evidence in this topic, different study designs, and different candidate genes selected in most of studies |
|  | 20d | Present results of all sensitivity analyses conducted to assess the robustness of the synthesized results. | Not applicable |
| **Reporting biases** | 21 | Present assessments of risk of bias due to missing results (arising from reporting biases) for each synthesis assessed. | Yes, risk bias assessment page 4 and supplementary Tables 5, 6, and 7 |
| **Certainty of evidence** | 22 | Present assessments of certainty (or confidence) in the body of evidence for each outcome assessed. | Risk of bias might reflect validity and reliability of the outcomes in the 15 studies included in this review article |
| **DISCUSSION** |  |  |  |
| **Discussion** | 23a | Provide a general interpretation of the results in the context of other evidence. | Yes, pages 7-10 |
|  | 23b | Discuss any limitations of the evidence included in the review. | Yes, pages 7-10 |
|  | 23c | Discuss any limitations of the review processes used. | Yes, page 10 |
|  | 23d | Discuss implications of the results for practice, policy, and future research. | Yes, pages 6-10. Sections Future directions and Conslusions |
| **OTHER INFORMATION** |  |  |  |
| **Registration and protocol** | 24a | Provide registration information for the review, including register name and registration number, or state that the review was not registered. | Yes. Page 2. The review protocol was registered in the International Prospective Register of Systematic Reviews (PROSPERO) with the reference number: CRD42021235431. |
|  | 24b | Indicate where the review protocol can be accessed, or state that a protocol was not prepared. | The review protocol is register in PROSPERO. reference number: CRD42021235431, https://www.crd.york.ac.uk/prospero/ |
|  | 24c | Describe and explain any amendments to information provided at registration or in the protocol. | Not applicable. The review protocol can be updated in PROSPERO database after publish the manuscript |
| **Support** | 25 | Describe sources of financial or non-financial support for the review, and the role of the funders or sponsors in the review. | Yes. Page 11 |
| **Competing interests** | 26 | Declare any competing interests of review authors. | Yes. Page 14 |
| **Availability of data, code and other materials** | 27 | Report which of the following are publicly available and where they can be found: template data collection forms; data extracted from included studies; data used for all analyses; analytic code; any other materials used in the review. | Supplementary files are publicly available, i.e., Table S1. Search terms used in PubMed, Web of Science, and Scopus databases. Table S2. PRISMA checklist 2020 Table S3. Risk of bias assessment of included cross-sectional studies. Table S4. Risk of bias assessment of acute physical activity studies. Table S5. Risk of bias assessment of included chronic physical activity studies (non-RCTs). |
