# Supplementary material for: Transcriptional and Epigenetic Response to Sedentary Behavior and Physical Activity in Children and Adolescents: A Systematic Review
Source: Front Pediatr. 2022 Jun 24;10:917152. doi: 10.3389/fped.2022.917152 (PMC9263076; doi:10.3389/fped.2022.917152)
Supplement: Supplementary file 4 [file Table_4.DOCX]

**Table S4.** Risk of bias assessment of included cross-sectional studies.

| **Study** | **Item 1** | **Item 2** | **Item 3** | **Item 4** | **Item 5** | **Item 6** | **Item 7** | **Item 8** |
| --- | --- | --- | --- | --- | --- | --- | --- | --- |
| Wu *et al.* 2015 (22) | NO | YES | NO | NA | YES | YES | YES | YES |
| Lovinsky-Desir *et al.* 2017 (23) | YES | YES | YES | NA | YES | YES | YES | YES |
| Vriens *et al.* 2018 (25) | NO | YES | NO | NA | YES | YES | YES | YES |
| Wu *et al.* 2020 (24) | NO | YES | YES | NA | YES | YES | YES | YES |
| Gopalan *et al.* 2020 (26) | YES | YES | NO | NA | NO | NO | YES | NO |
| Dos Santos Haber *et al.* 2022 (27) | YES | YES | NO | NA | YES | YES | YES | YES |
| Quality score per item % | 50% | 100% | 33.3% | NA | 83.3% | 83.3% | 100% | 83.3% |

The quality score per item (%) was calculated by dividing the number of studies that met the quality criteria in one specific item (e.g., answer as yes in item number 1) by the total number of studies (i.e., 6). The response “NA” was not considered to calculate the quality score per item (%). The lower is the score in each item (expressed in %) the lower is the quality of that item and therefore the higher is the bias in that item (e.g., 50 % in item number 1 and 100% in number 2 show a higher bias in item 1 compared to item number 2). YES: meet the quality criterion; NO: not meet the quality criterion; NA: Not applicable criterion.

The risk of bias assessment was performed using the Joanna Briggs Institute Critical Appraisal Tool for Systematic Reviews. The checklists used for cross-sectional studies include eight items. Item 1: were the criteria for inclusion in the sample clearly defined?; item 2: were the study subjects and the setting described in detail?; item 3: Was the exposure measured in a valid and reliable way?; item 4: were objective, standard criteria used for measurement of the condition?; item 5: were confounding factors identified?; item 6: were strategies to deal with confounding factors stated?; item 7: were the outcomes measured in a valid and reliable way?; item 8: was appropriate statistical analysis used?
