# Supplementary material for: Transcriptional and Epigenetic Response to Sedentary Behavior and Physical Activity in Children and Adolescents: A Systematic Review
Source: Front Pediatr. 2022 Jun 24;10:917152. doi: 10.3389/fped.2022.917152 (PMC9263076; doi:10.3389/fped.2022.917152)
Supplement: Supplementary file 5 [file Table_5.DOCX]

**Table S5.** Risk of bias assessment of acute physical activity studies (i.e., acute effects).

| Study | Item 1 | Item 2 | Item 3 | Item 4 | Item 5 | Item 6 | Item 7 | Item 8 | Item 9 | Item 10 | Item 11 | Item 12 | Item 13 | Item 14 | Item 15 | Item 16 | Item 17 |
| --- | --- | --- | --- | --- | --- | --- | --- | --- | --- | --- | --- | --- | --- | --- | --- | --- | --- |
| Radom-Aizik *et al.* 2009 (30) | YES | YES | YES | YES | YES | YES | YES | YES | YES | YES | YES | YES | YES | YES | YES | NO | YES |
| Radom-Aizik *et al.* 2009 (31) | YES | YES | YES | YES | YES | YES | YES | YES | YES | YES | YES | YES | YES | YES | YES | NO | YES |
| Kochanska-Dziurowicz et al. 2013 (29) | YES | YES | YES | YES | NO | YES | YES | YES | YES | YES | YES | YES | YES | YES | YES | NO | NO |
| Kilian et al. 2016 (28) | YES | YES | YES | YES | YES | YES | YES | YES | YES | YES | YES | YES | YES | YES | YES | YES | YES |
| Lu et al. 2017 (32) | YES | YES | YES | YES | YES | YES | YES | YES | YES | YES | YES | YES | YES | YES | YES | NO | NO |
| Quality score per item % | 100% | 100% | 100% | 100% | 80% | 100% | 100% | 100% | 100% | 100% | 100% | 100% | 100% | 100% | 100% | 20% | 60% |

The quality score per item (%) was calculated by dividing the number of studies that met the quality in one specific item (e.g., answer as yes in item number 1) by the total number of studies (i.e., 5). The lower is the score in each item (expressed in %) the lower is the quality of that item and therefore the higher is the bias in that item (e.g., 100% in item number 1 and 20% in number 16 show a higher bias in item 16 compared to item number 1). YES: meet the quality criterion; NO: not meet the quality criterion; NA: Not applicable criterion.

The risk of bias assessment was performed using a modified version of the Downs and Black checklist. The checklists used for acute PA studies include seventeen items. Item 1: is the hypothesis/aim/objective of the study clearly described?; item 2: Are the main outcomes to be measured clearly described in the introduction or methods section?; item 3: are the characteristics (e.g., age, height, weight, training and health status) of the participants included in the study clearly described?; item 4: are the interventions of interest clearly described?; item 5: are the main findings of the study clearly described?; item 6: does the study provide estimates of the random variability in the data for the main outcomes?; item 7: have all important adverse events that may be a consequence of the intervention been reported?, answer yes if they confirm they have ethical approval; item 8: was an attempt made to blind study subjects to the intervention they have received? for exercise interventions where it is not possible to blind, answer yes. Answer no if the intervention contains a supplement/placebo arm, and this is not blinded; item 9: was an attempt made to blind those measuring the main outcomes of the intervention?; item 10: if any of the results of the study were based on ‘data dredging’ was this made clear? Any analyses that had not been planned at the outset should be clearly indicated; item 11: was the timing of blood sampling clearly described?; item 12: were the statistical tests used to assess the main outcomes appropriate?; item 13: were the main outcome measures used accurate (valid and reliable)?; item 14: were study subjects randomised to intervention groups? Answer yes if the order of treatment, or allocation to groups, was randomly assigned. If it was not possible for the study to be randomised (e.g., single-trial studies) answer yes; item 15: was at least one familiarization session conducted prior to exercise testing?, answer yes if they conducted a familiarization trial, or if familiarization was not necessary (e.g., if the study uses a single, non-performance-based, exercise bout); item 16: were the exercise test conditions adequately standardised and described? factors to consider include confirmation of the time of day that testing was conducted, and control for unusual activity or nutritional factors in the days prior to the exercise test; item 17: was nutritional status for blood sampling adequately described?
