# Supplementary material for: Transcriptional and Epigenetic Response to Sedentary Behavior and Physical Activity in Children and Adolescents: A Systematic Review
Source: Front Pediatr. 2022 Jun 24;10:917152. doi: 10.3389/fped.2022.917152 (PMC9263076; doi:10.3389/fped.2022.917152)
Supplement: Supplementary file 6 [file Table_6.DOCX]

**Table S6.** Risk of bias assessment of included long-term physical activity interventions studies (i.e., chronic effects).

| **Study** | **Item 1** | **Item 2** | **Item 3** | **Item 4** | **Item 5** | **Item 6** | **Item7** | **Item 8** | **Item 9** |
| --- | --- | --- | --- | --- | --- | --- | --- | --- | --- |
| Lu et al. 2017 (32) | YES | YES | NA | NO | NA | YES | YES | YES | YES |
| Woo et al. 2011 (33) | YES | NA | NA | NO | NA | NO | YES | YES | NO |
| Blüher et al. 2019 (35) | YES | YES | NA | NO | NA | YES | YES | YES | YES |
| Zhao et al. 2021 (34) | YES | YES | NA | YES | NA | YES | YES | YES | NO |
| De Souza E Silva S et al. (36) | YES | YES | NA | YES | NA | YES | YES | YES | YES |
| Bias per item score % | 100% | 100% | NA | 40% | NA | 80% | 100% | 100% | 60% |

The quality score per item (%) was calculated by dividing the number of studies that met the quality in one specific item (e.g., answer as yes in item number 1) by the total number of studies (i.e., 5). The response “NA” was not considered to calculate the quality score per item score. The lower is the score in each item (expressed in %) the lower is the quality of that item and therefore the higher is the bias in that item (e.g., 100 % in item number 1 and 0% in number 4 show a higher bias in item 4 compared to item number 1). YES: meet the quality criterion; NO: not meet the quality criterion; NA: Not applicable criterion.

The risk of bias assessment was performed using the Joanna Briggs Institute Critical Appraisal Tool for Systematic Reviews. The checklists used for chronic exercise studies include nine items. Item 1: is it clear in the study what is the ‘cause’ and what is the ‘effect’ (i.e. there is no confusion about which variable comes first)?; item 2: were the participants included in any comparisons similar?; item 3: were the participants included in any comparisons receiving similar treatment/care, other than the exposure or intervention of interest?; item 4: was there a control group?; item 5: were there multiple measurements of the outcome both pre and post the intervention/exposure?; item 6: was follow up complete and if not, were differences between groups in terms of their follow up adequately described and analyzed?; item 7: were the outcomes of participants included in any comparisons measured in the same way?; item 8: were outcomes measured in a reliable way?; item 9: was appropriate statistical analysis used
